# Supplementary material for: Drug repurposing for aging research using model organisms
Source: Aging Cell. 2017 Jun 16;16(5):1006–15. doi: 10.1111/acel.12626 (PMC5595691; doi:10.1111/acel.12626)
Supplement: Supplementary file 7 — Data S1 Zip‐Archive of all report cards. [file ACEL-16-1006-s007.zip › RC_TYL.pdf]

TYL

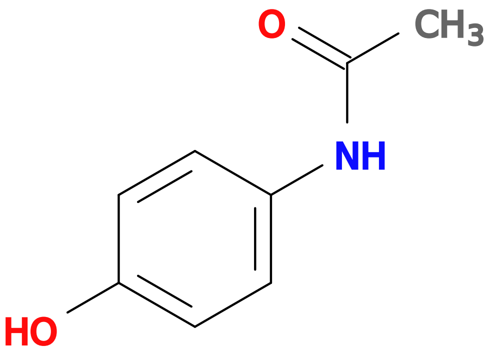

Database identifiers

ChEMBLCompound	CHEMBL112
DrugBank	DB00316
CHEBI	46195
ZINC	ZINC18274777
eMolecules	27677450
eMolecules	474380

Ranking

	Rank	Score
Drosophila	522/697	0.234
C. elegans	285/591	0.2

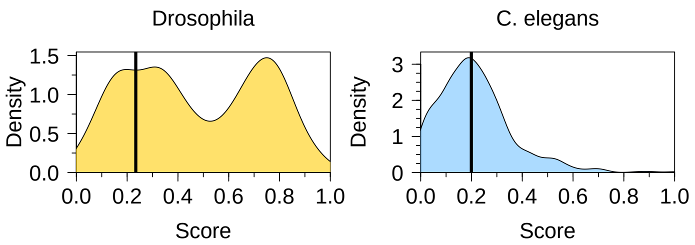

	Ageing implication	Domain conservation	Binding site conservation	Binding affinity	Bioavailability	Lipinski	Promiscuity	Purchasability	Drug approval	**Total**
Drosophila	0.203	0.597	0.877	0.362	(0.9)	0.0	-0.0	0.1	0.1	0.234
C. elegans	0.203	0.005	0.0	0.362	0.317	0.0	-0.0	0.1	0.1	0.2

Names

- **paracetamol**
- 4-(Acetylamino)phenol
- Acenol
- Acephen
- acetaminofen
- acetaminophene
- APAP
- Datril
- Infants' Feverall
- Injectapap
- Neopap
- Ofirmev

· p-acetaminophenol
· p-hydroxyacetanilide
· p-hydroxyphenolacetamide
· Phenaphen
· Tylenol

Roles

cyclooxygenase 2 inhibitor, cyclooxygenase 1 inhibitor, non-narcotic analgesic, antipyretic, non-steroidal anti-inflammatory drug, xenobiotic, human blood serum metabolite, cyclooxygenase 3 inhibitor, environmental contaminant, Cyclooxygenase inhibitor, Anandamide amidohydrolase inhibitor, Vanilloid receptor opener

Status

Approved drug (according to ChEMBL)	Yes
Administration Route	Oral, Rectal
Number of Rule of 5 violations	0
Binding affinity to original target in log units (RF-Score prediction)	4.43
Burns *C. elegans* bioavailability prediction	0.04

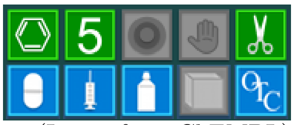

(Image from ChEMBL)

Medical Information

Indicatation: For temporary relief of fever, minor aches, and pains.
Mechanism of action: Acetaminophen is thought to act primarily in the CNS, increasing the pain threshold by inhibiting both isoforms of cyclooxygenase, COX-1, COX-2, and COX-3 enzymes involved in prostaglandin (PG) synthesis. Unlike NSAIDs, acetaminophen does not inhibit cyclooxygenase in peripheral tissues and, thus, has no peripheral anti-inflammatory affects. While aspirin acts as an irreversible inhibitor of COX and directly blocks the enzyme's active site, studies have found that acetaminophen indirectly blocks COX, and that this blockade is ineffective in the presence of peroxides. This might explain why acetaminophen is effective in the central nervous system and in endothelial cells but not in platelets and immune cells which have high levels of peroxides. Studies also report data suggesting that acetaminophen selectively blocks a variant of the COX enzyme that is different from the known variants COX-1 and COX-2. This enzyme is now referred to as COX-3. Its exact mechanism of action is still poorly understood, but future research may provide further insight into how it works. The antipyretic properties of acetaminophen are likely due to direct effects on the heat-regulating centres of the hypothalamus resulting in peripheral vasodilation, sweating and hence heat dissipation.
Toxicity: Oral, mouse: LD50 = 338 mg/kg; Oral, rat: LD50 = 1944 mg/kg. Acetaminophen is metabolized primarily in the liver, where most of it is converted to inactive compounds by conjugation with glucuronic acid and, to a lesser extent, sulfuric acid. Conjugates are then excreted by the kidneys. Only a small portion is excreted in unchanged in urine or oxidized via the hepatic cytochrome P450 enzyme system (CYP2E1). Metabolism via CYP2E1 produces a toxic metabolite, N-acetyl-p-benzoquinoneimine (NAPQI). The toxic effects of acetaminophen are due to NAPQI, not acetaminophen itself nor any of the major metabolites. At therapeutic doses, NAPQI reacts with the sulfhydryl group of glutathione to produce a non-toxic conjugate that is excreted by the kidneys. High doses of acetaminophen may cause glutathione depletion, accumulation of NAPQI and hepatic necrosis. The maximum daily dose of acetaminophen is 4 g. Liver failure has been observed at doses as low as 6 g per day. As such, the maximum daily and single dose of acetaminophen is currently being reviewed in some countries. N-acetyl-cysteine, a precursor of glutathione, may be administered in the event of acetaminophen toxicity.
Metabolism: Acetaminophen primarily undergoes glucuronidation (45-55% of the dose) in which

this process is facilitated by UGT1A1, UGT1A6, UGT1A9, UGT2B15 in the liver or UGT1A10 in the gut. 30-35% of the dose undergoes sulfation. This biotransformation is facilitated by SULT1A1, SULT1A3, SULT1A4, SULT1E1 and SULT2A1. A small percentage of acetaminophen is oxidized by CYP2E1 to form N-acetyl-p-benzo-quinone imine (NAPQI), a toxic metabolite which is then conjugated to glutathione and excreted renally. Studies suggest that CYP3A4 and CYP2E1 are the primary cytochrome P450 isozymes responsible for the generation of toxic metabolites. Accumulation of NAPQI may occur if primary metabolic pathways are saturated.
Retail: Actavis Group, Advent Pharmaceuticals Inc., Amneal Pharmaceuticals, Aristos Pharmaceuticals, A-S Medication Solutions LLC, Bergen Brunswig and 37 more suppliers
Tencon tablet - 1.01 USD / tablet
Tylenol with Codeine 3 300-30 mg tablet - 1.1 USD / tablet
Hydrocodone-apap 10-750 tablet - 1.12 USD / tablet
Darvocet-n 50 tablet - 1.14 USD / tablet
Norco 5-325 tablet - 1.19 USD / tablet
Phrenilin tablet - 1.32 USD / tablet
and 167 more options

(Information from DrugBank)

Compound Target Characteristics

CREB-binding protein

Best gene implication in ageing for this target family came from gene Q92793 via mapping the annotation from Ensembl ENSG00000005339 via mapping the annotation from EntrezGene 1387 via mapping the annotation from GenAgeHuman 0064 annotated in GenAge release 17.

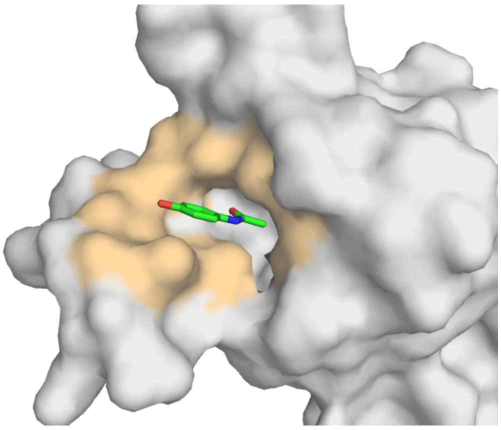

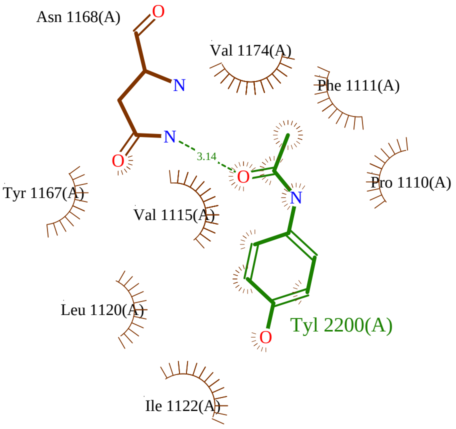

protein	amino acids contacts (binding site)							
PDB:4a9k:chainA:Q92793	P	F	V	L	I	Y	N	V
sp:Q09472:EP300_HUMAN	P	F	V	L	I	Y	N	V
sp:Q92793:CBP_HUMAN	P	F	V	L	I	Y	N	V
tr:F1M9G7:F1M9G7_RAT	P	F	V	L	I	Y	N	V
tr:Q8QZV8:Q8QZV8_MOUSE	P	F	V	L	I	Y	N	V
tr:F8VPR5:F8VPR5_MOUSE	P	F	V	L	I	Y	N	V
tr:Q8T3Z6:Q8T3Z6_DROME	H	F	V	L	V	Y	N	V
tr:Q058W2:Q058W2_DROME	H	F	V	L	V	Y	N	V
tr:Q9VCG6:Q9VCG6_DROME	H	F	V	L	V	Y	N	V
tr:O17681:O17681_CAEEL	-	-	-	-	-	-	-	-
tr:Q9TZB8:Q9TZB8_CAEEL	-	-	-	-	-	-	-	-
sp:Q07442:BDF2_YEAST	P	F	V	L	L	F	N	V

protein	whole protein ident	simil	domain-based ident	simil	contact-based ident	simil
PDB:4a9k:chainA:Q92793	0.97	0.99	1.0	1.0	1.0	1.0
sp:Q09472:EP300_HUMAN	0.59	0.81	0.86	0.96	1.0	1.0
sp:Q92793:CBP_HUMAN	1.0	1.0	1.0	1.0	1.0	1.0
tr:F1M9G7:F1M9G7_RAT	0.96	0.99	0.98	0.99	1.0	1.0
tr:Q8QZV8:Q8QZV8_MOUSE	0.95	0.98	0.98	1.0	1.0	1.0
tr:F8VPR5:F8VPR5_MOUSE	0.96	0.99	0.98	1.0	1.0	1.0
tr:Q8T3Z6:Q8T3Z6_DROME	0.02	0.06	0.29	0.64	0.75	0.88
tr:Q058W2:Q058W2_DROME	0.02	0.09	0.28	0.63	0.75	0.88
tr:Q9VCG6:Q9VCG6_DROME	0.03	0.12	0.29	0.64	0.75	0.88
tr:O17681:O17681_CAEEL	0.01	0.04	0.0	0.0	0.0	0.0
tr:Q9TZB8:Q9TZB8_CAEEL	0.03	0.07	0.0	0.0	0.0	0.0
sp:Q07442:BDF2_YEAST	0.03	0.15	0.29	0.7	0.75	0.97

tbrd-1 (FBgn0039124) associated phenotypes

male fertile

(Information from FlyBase)
